# Prospective Pilot Clinical Study of High-Purity Magnesium Plates and Screws for Le Fort I Osteosynthesis

**DOI:** 10.1016/j.identj.2026.109644

**Published:** 2026-05-25

**Authors:** Wei-fa Yang, Pak Chuen Patrick Tai, Ling Qin, Yiu Yan Leung

**Affiliations:** a Division of Oral and Maxillofacial Surgery, Faculty of Dentistry, The University of Hong Kong; Prince Philip Dental Hospital, Hong Kong, China; b Department of Orthopaedics & Traumatology and Innovative Orthopaedic Biomaterial & Drug Translational Research Laboratory, Li Ka Shing Institute of Health Sciences, The Chinese University of Hong Kong, Hong Kong, China

**Keywords:** High-purity magnesium, Orthognathic surgery, Jaw surgery, Biodegradable, Maxilla, Internal fixation

## Abstract

**Objectives:**

This prospective clinical study aimed to evaluate the feasibility, safety, and clinical performance of an innovative high-purity magnesium (HP-Mg) plate and screw system for internal fixation in orthognathic surgery.

**Materials and Methods:**

Patients indicated for orthognathic surgery fulfilling the study criteria were invited to participate. HP-Mg plates and screws were developed for the first time for Le Fort I fixation in the maxilla, while standard titanium fixation was used in the mandible for comparison. Clinical outcomes, wound healing, neurosensory function, radiographic imaging, systemic complications, and plasma electrolyte levels were assessed over a one-year follow-up.

**Results:**

Seven patients were included. HP-Mg plates and screws were systemically safe, with no evidence of hypermagnesemia or related neurosensory impairment. Two patients experienced smooth recovery; 3 developed mild complications (e.g., wound dehiscence, fistula) that were managed conservatively; and 2 required reoperations due to maxillary nonunion. Radiographically, HP-Mg plates and screws were not visible on plain X-rays but were detectable on CBCT, exhibiting reduced radiodensity compared to cortical bone. The complications were attributed to unoptimized plate geometry and rapid *in vivo* degradation.

**Conclusions:**

Although HP-Mg plates and screws represent a promising biodegradable alternative for internal fixation in orthognathic surgery, the observed complications highlight opportunities for further optimization—particularly in enhancing degradation profiles and mechanical strength. With ongoing research and continued improvements, Mg has the potential to become a reliable and effective option for internal fixation in maxillofacial surgery.

**Clinical Relevance:**

While HP-Mg is a promising biodegradable alternative for internal fixation in orthognathic surgery, further optimization of implant design and degradation profiles is necessary.

## Introduction

Orthognathic surgery is a corrective procedure designed to address jaw deformities, with plate and screw fixation playing a crucial role in maintaining stability during bone healing.^1^ Titanium and its alloys are considered the gold standard for fixation devices due to their excellent mechanical strength and biocompatibility. However, the rigidity of titanium significantly exceeds that of natural bone, which can result in stress shielding and subsequent bone resorption, particularly in load-bearing conditions. Furthermore, because these fixation devices are intended as temporary mechanical supports, a second surgery is sometimes required for their removal, increasing both patient morbidity and healthcare costs.^2^^,^^3^ If left *in situ*, these titanium hardware components may also complicate radiographic imaging, as they can cause beam-hardening artifacts that obscure diagnostic details.

To address these limitations, bioresorbable fixation systems have been developed as alternative solutions. Typically composed of polymers such as poly(lactic acid) (PLA), polyglycolic acid (PGA), or their copolymers (PLGA), these systems gradually degrade in the body, thereby eliminating the need for a secondary procedure to remove the hardware.^4^ However, their relatively low mechanical strength often necessitates the use of bulkier implants, which can hinder surgical handling and shape adaptation. In addition, their biocompatibility is suboptimal, with the potential to induce foreign body reactions.^5^ Furthermore, unpredictable degradation rates pose additional risks for complications such as malunion or nonunion of bone segments.

In recent years, magnesium (Mg) has emerged as a promising biodegradable metal for osteosynthesis,^6^, ^7^, ^8^ offering mechanical properties more closely aligned with those of native bone than titanium or stainless steel. This similarity helps minimize both stress shielding and interference with radiographic imaging. In vivo, Mg implants gradually degrade, releasing Mg ions—vital for numerous physiological processes—and hydrogen gas, which typically dissipates without causing long-term adverse effects.^9^ Notably, Mg ions have demonstrated osteogenic potential, promoting bone regeneration in both preclinical and clinical studies.^6^^,^^8^, ^9^, ^10^, ^11^ Collectively, these advantages position Mg as a leading candidate for next-generation biodegradable internal fixation materials.

The clinical application of Mg in bone surgery dates back to 1906.^12^ Recent advancements have led to the development of commercial implants such as MAGNEZIX® (Mg-Y-Re-Zr alloy) and RESOMET™ (Mg-Ca-Zn alloy), which are used for indications including hallux valgus and distal radius fractures.^12^ However, concerns about the biocompatibility of certain alloying elements have driven research toward high-purity magnesium (HP-Mg) implants.^11^^,^^13^, ^14^, ^15^, ^16^, ^17^, ^18^, ^19^ In China, clinical trials with HP-Mg screws have shown promising results for conditions such as ankle fractures, femoral neck fractures, osteonecrosis of the femoral head, and trauma-induced avascular necrosis.^7^^,^^20^, ^21^, ^22^ These studies reported no adverse effects from degradation products, and plasma Mg levels remained within the normal physiological range.

Despite its potential, HP-Mg faces challenges in orthopaedics due to its limited mechanical strength in weight-bearing areas. For example, hip joint fixation must withstand forces over 2000 N—about 3 times body weight—which can increase further with mobilization.^23^ To address these limitations, researchers are developing coatings to slow Mg degradation and employing metallurgical techniques to enhance its strength.^24^^,^^25^ In contrast, jaw surgeries require lower force resistance, with normal occlusal forces around 400 N, dropping to 115 N at one week and 250 N at 6 weeks post-operation.^26^ Therefore, HP-Mg shows particular promise for use in jaw surgery.

However, clinical evidence for Mg-based fixation in jaw surgery is still lacking.^27^ This prospective clinical study aims to innovatively assess the feasibility and outcomes of HP-Mg plate and screw fixation in orthognathic surgery, with the goal of developing optimized surgical protocols. By addressing current knowledge gaps, this research may promote wider adoption of biodegradable fixation devices in jaw surgery, ultimately improving patient outcomes and reducing healthcare costs.

## Materials and methods

### High-purity magnesium plates and screws

**Fig. 1:**
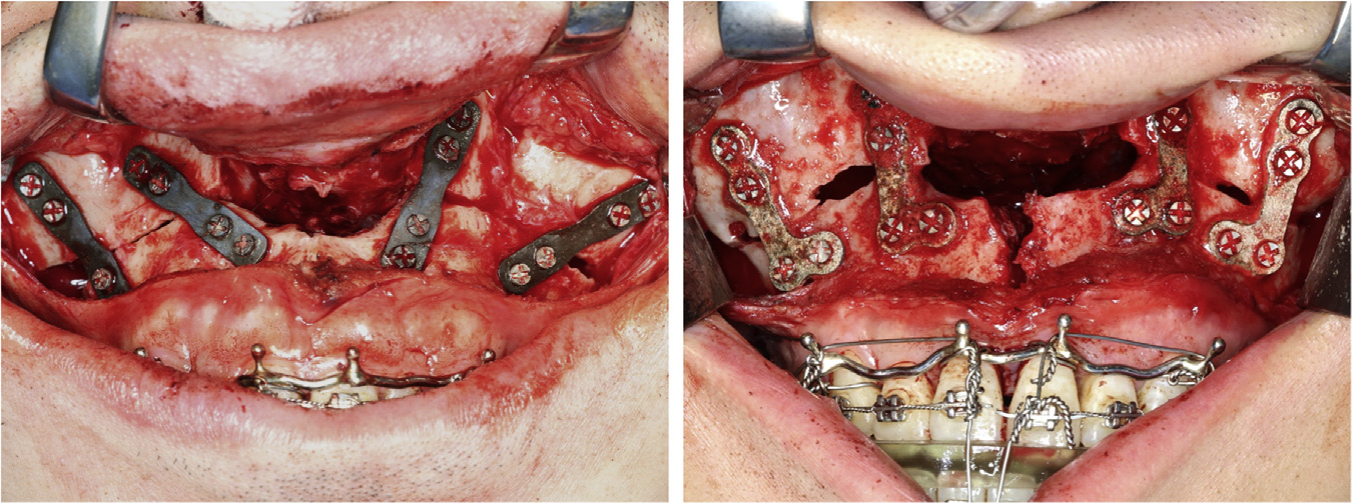
The magnesium plates featured a universal 4-hole straight (left) or L-shaped (right) design. The magnesium plates and screws were designed based on the geometry of the commercial titanium system MatrixORTHOGNATHIC (DePuy Synthes, Johnson & Johnson Medtech).

The HP-Mg plates and screws were designed based on geometry of the MatrixORTHOGNATHIC (DePuy Synthes, Johnson & Johnson Medtech, US) titanium system commonly used in orthognathic surgery. The HP-Mg plates featured a universal 4-hole straight or L-shaped design, measuring 5.5 mm in width and 1 mm in thickness, with 2.3 mm screw holes. (Figure 1) The Mg screws measured 8 mm in length (including the head) and 1.85 mm in diameter, ensuring compatibility with the surgical drilling instruments used in our operation theatre. These plates and screws were manufactured from HP-Mg (99.99 wt%) at Dongguan Eontec Co., Ltd. (Guangdong, China), using extrusion and CNC machining techniques.^28^ The manufacturing process complied with GMP standards and was certified under ISO 13485:2016 for the design, development, manufacture, marketing, and service of degradable Mg bone internal fixation screws. Certified test results showed that the HP-Mg screws had a tensile strength of 150 MPa and a yield strength of 50 MPa, making these screws suitable for use as biodegradable bone screws in surgery. After production, the plates and screws were cleaned, individually packaged, and sterilized using high-energy electron beam irradiation, making them ready for clinical application.

### Study design

This prospective, single-arm interventional clinical study was designed to evaluate the efficacy and safety of HP-Mg plate and screw fixation in orthognathic surgery. Efficacy was defined as the rate of successful bone healing and intended functional recovery of the jaw after surgery. Safety was assessed with multiple metrics, including Mg degradation, local side effects, and systemic complications. The study was carried out in accordance with the ICH-GCP guidelines and in strict adherence to the principles outlined in the Declaration of Helsinki. The study protocol was reviewed and approved by the Institutional Review Board of The University of Hong Kong/Hospital Authority Hong Kong West Cluster (HKU/HA HKW IRB: UW 24-175). The study protocol was registered in ClinicalTrials.gov (NCT06536660).

### Subjects

Patients indicated for orthognathic surgery requiring internal fixation were invited to participate in this study. Eligible participants were adults aged 18 years or older with a confirmed indication for orthognathic surgery and adequate bone quality and volume to support the placement of Mg plates and screws. All participants were required to provide written informed consent after being informed of the potential risks and benefits associated with Mg fixation. Exclusion criteria included medical conditions that could impair bone healing or increase the risk of complications, such as uncontrolled diabetes, severe osteoporosis, multiple myeloma, or active bone infection. Patients with conditions that could exacerbate or predispose them to systemic side effects of Mg, including chronic kidney disease, hypermagnesemia, severe electrolyte imbalances, severe heart disease (especially those with a history of arrhythmias or heart failure), myasthenia gravis, abnormal thyroid function, hyperparathyroidism, or adrenocortical insufficiency, were also excluded. Additional exclusion criteria were known allergy or hypersensitivity to Mg, an ASA score of 3 or higher, pregnancy or breastfeeding, and any circumstances likely to affect compliance with the study protocol, such as inability to attend follow-up visits or adhere to post-operative care instructions.

### Surgery

Patients enrolled in the study underwent orthognathic surgery to correct skeletal disharmonies, imbalances, asymmetries, and disproportions. Comprehensive preoperative preparation was performed in all cases, including model surgery and computer-assisted simulation to optimize surgical planning and jaw movement. Intraoperatively, vestibular incisions were made, followed by standard osteotomy techniques such as Le Fort I (1-piece or 2-piece) osteotomies of the maxilla and bilateral sagittal split osteotomies of the mandible. After completion of the osteotomies, the tooth-bearing segments were fully mobilized and repositioned to achieve optimal functional and aesthetic outcomes. The maxilla was stabilized using HP-Mg plates and screws placed along the ideal lines of osteosynthesis, while the mandible or chin was fixed using the conventional titanium system. Standard surgical protocols and perioperative management were strictly followed. Postoperatively, light training elastics (RZJZGZ Orthodontic Elastic Bands Monkey 2/8’’) were placed. Patients gradually progressed from a liquid or soft diet to a normal diet as tolerated. All surgeries were performed by the same surgical team under the supervision of a single consultant surgeon. We adhered to the standard surgical techniques, plate positioning protocols, and perioperative management practices established at our centre.

### Clinical assessment

Perioperative clinical assessments were performed routinely, with particular emphasis on the postoperative period to ensure optimal healing and recovery. Patients were followed up at 1 week, 3 weeks, 6 weeks, 3 months, 6 months, and 1 year after surgery. At each follow-up visit, in addition to standard inquiries regarding patient complaints and necessary wound care, comprehensive assessments were conducted to evaluate wound healing, functional recovery, and local side effects. Wound healing was assessed by visual inspection and palpation of the surgical site. Functional recovery was evaluated by monitoring occlusal rehabilitation and the patient's progression toward resuming a normal diet. Any local side effects, including emphysema, infection, implant rejection, screw loosening, or delayed healing, were documented and managed as appropriate.

### Radiographic examination

During postoperative follow-up visits, plain X-ray or cone-beam computed tomography (CBCT) imaging was routinely performed or obtained when clinically indicated. Bone structure was assessed using plain X-rays immediately after surgery and at 3 months, 6 months, and 1 year postoperatively. CBCT scans were also acquired to evaluate bone healing and potential complications immediately after surgery (as a baseline reference), as well as at 3 months and 1 year after surgery. Radiographic evaluations were conducted by experienced surgeons as part of routine postoperative management. Bone gap widths were measured for the comparison of bone healing. Mg hardware degradation was assessed by measuring changes in hardware volume over time. Gas retention was characterized by the presence and progression of hypo-intense areas adjacent to the Mg plates.

### Systemic side effects

Although Mg is an essential mineral for maintaining healthy muscles, nerves, bones, and blood sugar levels—and hypermagnesemia is a relatively uncommon abnormality—it was theoretically possible that abrupt release of Mg ions from hardware degradation could result in a transient increase in plasma Mg levels. Such elevations could induce systemic side effects, particularly in patients with predisposing conditions such as acute kidney injury, chronic kidney disease, or adrenocortical insufficiency.^29^ Clinical manifestations of hypermagnesemia, including bradycardia, hypotension, reduced consciousness, and respiratory depression, were closely monitored during the in-hospital stay and follow-up visits.^29^ Patients were educated about the signs and symptoms of potential systemic complications and encouraged to seek medical attention if any concerns arose. As abnormal Mg levels and their management could affect potassium and calcium homeostasis, plasma levels of Mg, calcium, and potassium, as well as liver and kidney function tests, were assessed during hospitalization and at follow-up appointments. Any abnormal findings were promptly investigated and managed as appropriate.

### Data analysis

All data collected during the study were entered into a secure database and well organized. Descriptive statistics and comparative analyses were performed where applicable. All data analysis and visualization were conducted using Microsoft Excel (Microsoft 365 MSO, Version 2506).

## Results

Seven consecutive patients were recruited and successfully underwent surgery with HP-Mg fixations, consisting of 6 males and one female, with a mean age of 28.4 ± 5.3 years. All patients received double jaw surgery. Personalized jaw movements were meticulously planned and achieved to address each patient’s specific conditions. Within the cohort, 2 patients were classified as overweight (BMI 25-30 kg/m²), while the remaining 5 had a normal BMI. Detailed information on the surgical information for each patient is provided in Table 1.

**Table 1:** Patient demographics and surgical information.

Case	Gender/Age	Height/Body Weight	Procedure	Maxilla movement	Mandible movement	Chin movement
A	M/24y	169 cm/68.3 kg	LeFort I, BSSO, genioplasty, removal 18, 28	Setback 3 mm, downgraft 1mm, clockwise rotation, decanting	Setback 7 mm (right), 10 mm (left)	-
B	M/24y	183 cm/67.9 kg	LeFort I, BSSO, mandibular contouring, removal 18, 28, 38	Advance 3 mm, downgraft 4 mm, clockwise rotation, decanting	Setback 18 mm (right), 8 mm (left)	-
C	M/26y	176 cm/70.6 kg	LeFort I, BSSO	Keep AP and vertical, decanting	Advance 3 mm (right), 7 mm (left)	-
D	M/39y	187 cm/99 kg	LeFort I, BSSO	Advance 2 mm, downgraft 2mm, clockwise rotation	Setback 15 mm (right), 11 mm (left)	-
E	M/26y	183 cm/76.5 kg	LeFort I x2, BSSO	Setback 3 mm, downgraft 2 mm, constrict 1mm	Setback 5 mm (right), 8 mm (left)	-
F	M/30y	174 cm/82.2 kg	LeFort I, BSSO, genioplasty, mandibular contouring	Advance 1 mm, downgraft 1 mm, clockwise rotation, decanting	Setback 2 mm (right), 9 mm (left)	Advance 5 mm
G	F/30y	164 cm/60.5 kg	Bilateral reduction malarplasty, LeFort I, BSSO, genioplasty	Reduce 3 mm (zygoma); Setback 3 mm, downgraft 3 mm, clockwise rotation	Setback 8.5 mm (right), 10 mm (left)	Constriction 6 mm

BSSO: bilateral sagittal split osteotomies of the mandible.

**Fig. 2:**
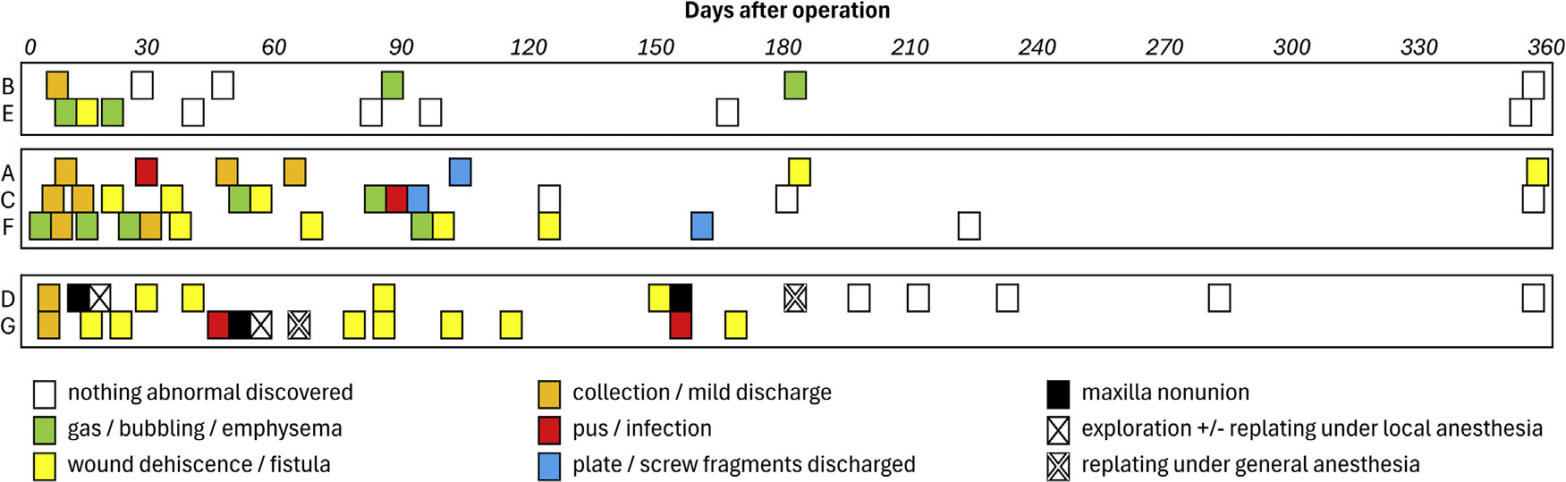
Postoperative events of 7 patients (A–G) who underwent Le Fort I osteotomy with magnesium fixation. Patients were classified into 3 categories based on clinical progression: Category 1 (B, E) had stable healing with minor gas retention; Category 2 (A, C, F) exhibited prolonged discharge until spontaneous ejection of plate/screw fragments and wound infection resolved; Category 3 (D, G) developed wound dehiscence and maxillary nonunion, requiring surgical reintervention.

**Fig. 3:**
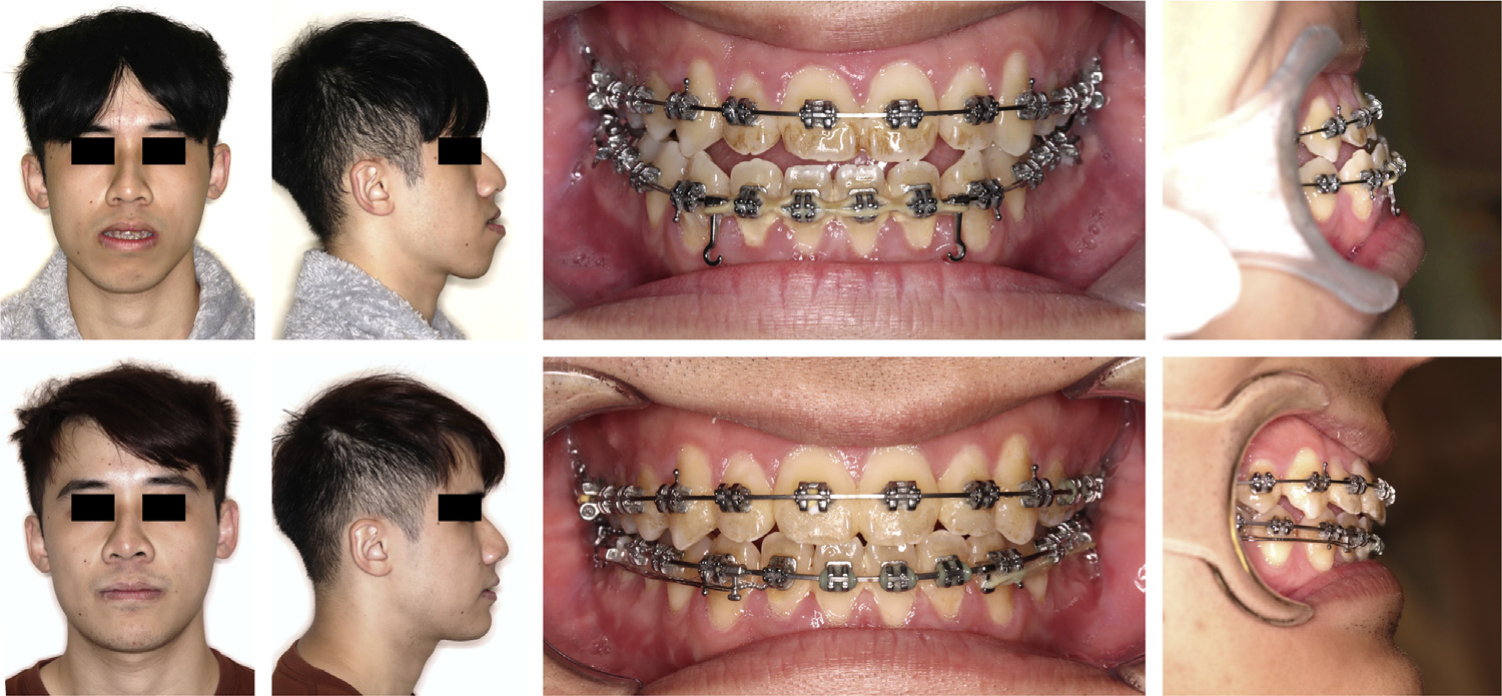
A case example (patient A) achieved ideal occlusal function and satisfactory aesthetic outcomes as planned. Upper row: before surgery; lower row: at 1 year after surgery.

The postoperative journey of the patients is illustrated in Figure 2. Among the 7 patients analysed, 2 (patients B and E) experienced uneventful healing, with only transient gas accumulation or emphysema, and demonstrated stable osseous integration and favourable postoperative outcomes. Three patients (A, C, and F) developed mild to moderate complications—including wound dehiscence, serous discharge, and occasional infection or plate/screw exposure—which were managed conservatively without the need for surgical intervention. The remaining 2 patients (D and G) encountered severe complications, specifically maxillary nonunion, ultimately requiring surgical re-exploration and replating under local or general anaesthesia. Despite these challenges, all patients achieved ideal occlusal function and satisfactory aesthetic outcomes as planned (Figure 3).

**Fig. 4:**
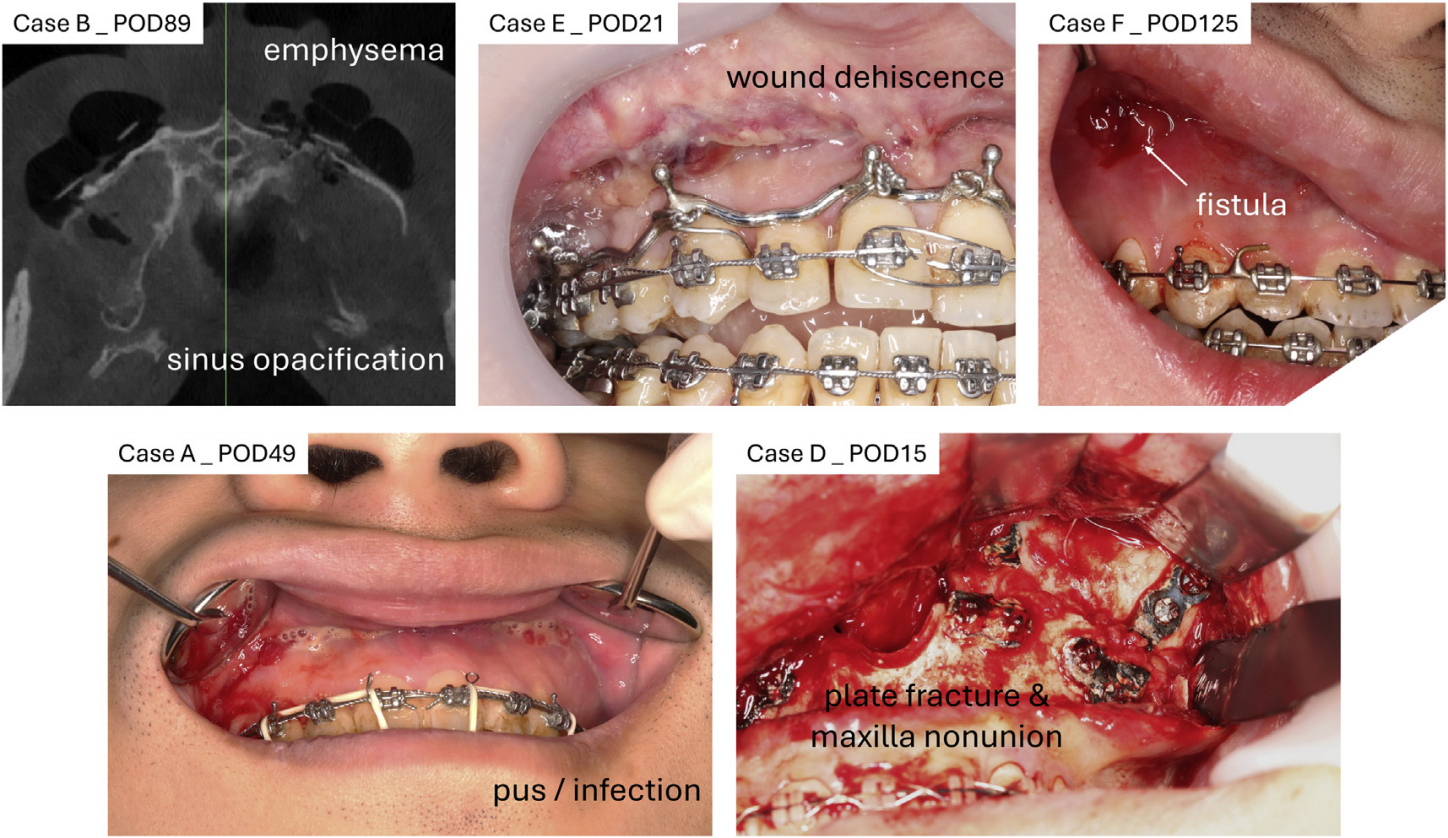
Surgical complications following Le Fort I osteotomy with magnesium fixation as revealed by clinical examination, radiographic assessment, and surgical exploration. Upper left: gas retention or emphysema shown in CBCT; upper middle: wound dehiscence; upper right: wound fistula; lower left: signs of infection with pus discharge; lower right: plate fracture and maxilla nonunion confirmed during surgical exploration. The occurrence of complications was documented in Figure 2 above.

To illustrate the spectrum of postoperative complications, representative clinical and radiographic images were selected (Figure 4). Commonly encountered issues during follow-up included subcutaneous emphysema and sinus opacification, as demonstrated on CT imaging. Soft tissue complications were also prevalent, with clinical photographs documenting wound dehiscence, mucosal fistula formation, and localized infection with pus discharge. In one photograph, intraoperative findings revealed plate fracture and maxillary nonunion.

**Fig. 5:**
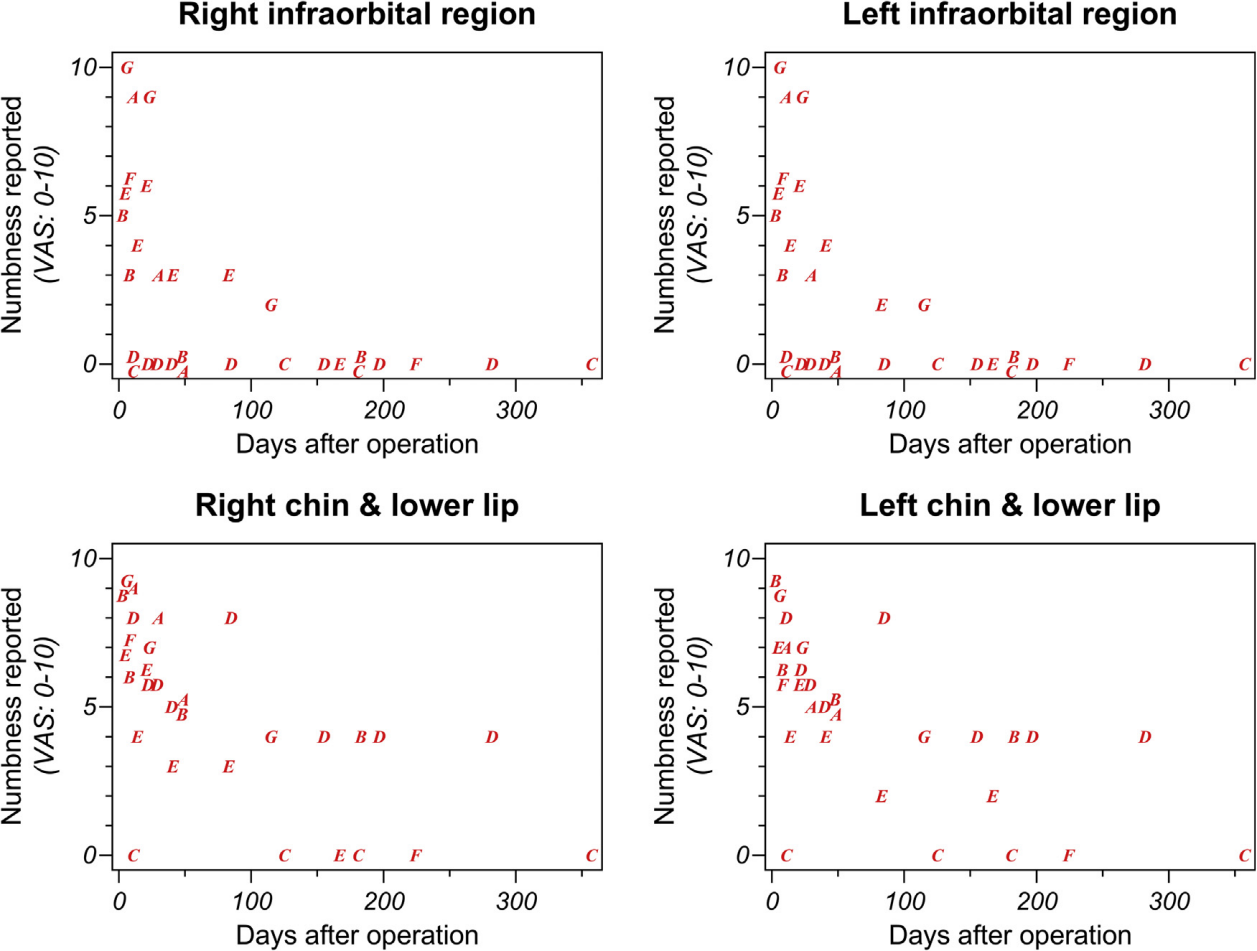
Postoperative neurosensory function was assessed using the visual analogue scale (VAS) in both the infraorbital and mental regions.

Postoperative neurosensory function was assessed using the visual analogue scale (VAS) in both the infraorbital and mental regions (Figure 5). Infraorbital numbness, where the HP-Mg plates were placed, was transient and demonstrated near-complete resolution within one year. No evidence of prolonged or worsening impairment attributable to Mg was observed.^30^ In contrast, numbness in the lower lip and chin was more persistent, with several patients experiencing moderate numbness that extended beyond 6 months. These findings suggest that Mg degradation did not exacerbate nerve injury in the infraorbital region following Le Fort I osteotomy.

**Fig. 6:**
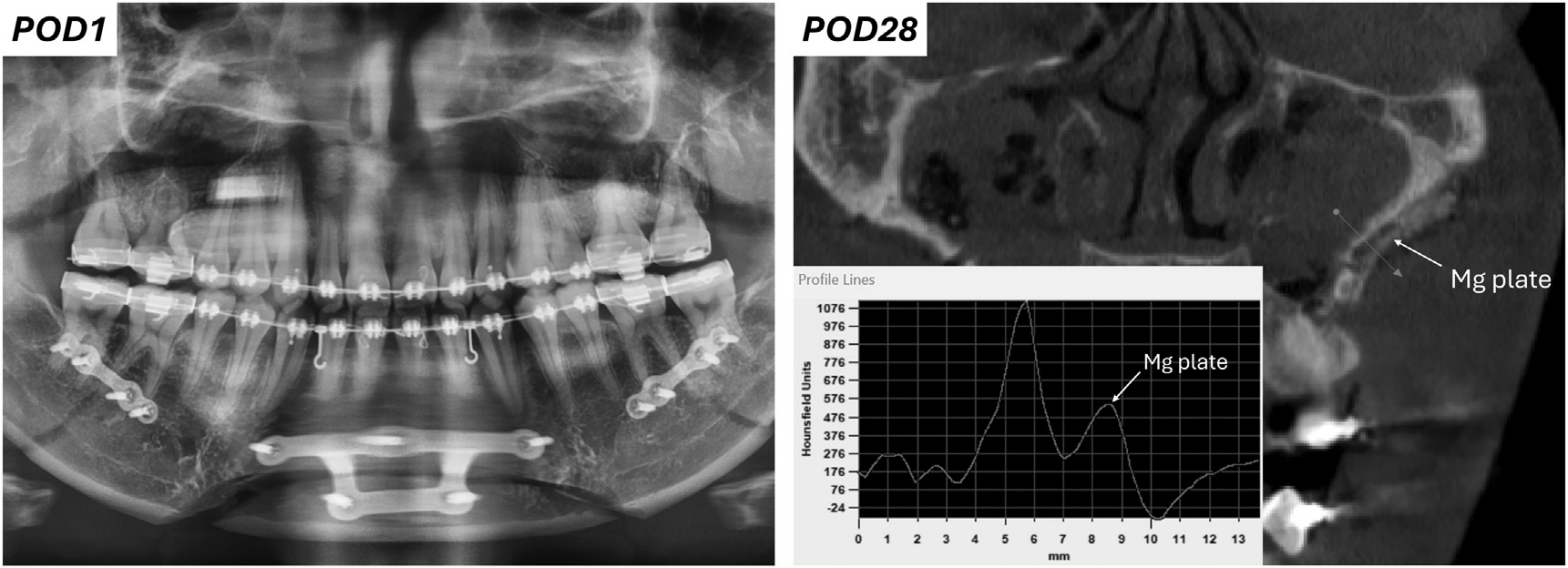
Representative postoperative imaging of magnesium fixation following Le Fort I osteotomy. Panoramic radiograph shows well-positioned maxilla, while magnesium plates and screws are invisible. CBCT confirms visible Mg plate along the lateral maxillary wall, while the radiodensity of Mg is lower than the cortical bone.

Representative postoperative radiographic images are shown in Figure 6. The HP-Mg plates and screws were not visible on panoramic radiography, in contrast to the titanium plates and screws in the mandible. CBCT was able to detect the Mg hardware. A focused analysis of Hounsfield units (HU) confirmed that the peak intensity of the Mg plate was noticeably lower than that of cortical bone.

**Fig. 7:**
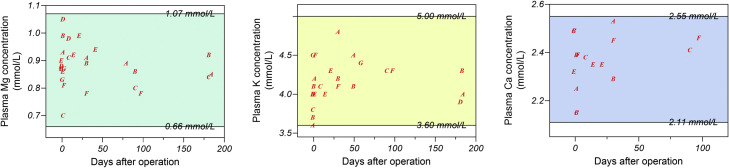
Plasma magnesium, potassium, and calcium levels remained within physiological limits throughout the follow-up period.

No systemic side effects were observed during the follow-up visits. Plasma Mg, potassium, and calcium levels remained within physiological ranges throughout the follow-up period (Figure 7), indicating that Mg degradation did not cause systemic toxicity or electrolyte imbalance.

## Discussion

This prospective clinical study investigated the feasibility, safety, and clinical outcomes of using HP-Mg plates and screws in Le Fort I osteosynthesis, representing a pioneering application of biodegradable metal fixation in maxillofacial surgery.^27^ Although HP-Mg has shown promising results in orthopaedic and trauma settings, its use in maxillofacial procedures—where anatomical complexity, microbial exposure, and masticatory forces present unique challenges—remains largely unexplored.^31^^,^^32^

Our findings indicate that HP-Mg is systemically safe. Postoperative neurosensory function was not adversely affected by Mg,^30^ and no cases of hypermagnesemia or electrolyte imbalance were observed. However, the rate of local complications was considerable. Two of 7 patients experienced fixation failure with nonunion, necessitating reoperation and replacement with titanium plates.^33^ Several others developed wound dehiscence, fistula formation, or infection.^34^, ^35^, ^36^, ^37^ These complications underscore significant mechanical and degradation-related challenges associated with HP-Mg in orthognathic surgery.

Unlike orthopaedic applications—such as intramedullary screws or fixation plates in long bones—maxillofacial fixation must withstand more complex 3-dimensional forces, including significant shearing stresses generated by functional jaw movements.^38^ In this study, we chose to first investigate the use of HP-Mg fixation in the maxilla, rather than the mandible, given its relatively static nature and lower functional loading during activities such as speaking, yawning, or mouth opening. Nevertheless, the clinical outcomes were still unsatisfactory, suggesting that inadequate load distribution and suboptimal mechanical strength may have contributed to early fixation failure. Notably, the screw and plate dimensions and geometry used in this study were adapted directly from the MatrixORTHOGNATHIC™ titanium system, without biomechanical optimization for Mg. The adoption of titanium plate geometry was a deliberate initial approach to minimize variables in this first-in-field study, allowing direct comparison of material performance without confounding differences in geometry. A bulkier design may provide greater mechanical strength and help overcome the hardware failures observed in this study, which warrants further investigation.

Moreover, the degradation kinetics of HP-Mg are a critical determinant of clinical success. Excessive or premature degradation can compromise mechanical stability before bone consolidation is achieved. In our cases, the HP-Mg hardware began to degrade upon contact with fluids—such as blood and irrigation solutions—during osteotomy or screw hole drilling, which is unavoidable in real surgery. We observed surface bubbles, erosion pits, and discoloration from white to darker hues^39^, raising concerns about early fixation weakening. Similar issues have been reported in preclinical studies, suggesting that uncoated HP-Mg may degrade too rapidly in high-fluid environments, thus undermining *in vivo* performance.^39^ Surface modifications—such as plasma electrolytic oxidation (PEO), physical vapor deposition (PVD), or the application of inorganic or polymer coatings—have been shown to enhance corrosion resistance and prolong mechanical integrity,^24^^,^^25^^,^^40^, ^41^, ^42^ and should be considered in future device iterations.

Beyond surface modifications, alloying strategies represent another important avenue for enhancing Mg-based implant performance. Commercial Mg-based systems such as MAGNEZIX® (Mg-Y-Re-Zr alloy) and RESOMET (Mg-Ca-Zn alloy) have demonstrated improved mechanical properties and controlled degradation profiles. Mg-Al and Mg-Zn alloy systems have been extensively studied, with the addition of elements such as zinc, calcium, manganese, and rare earth metals potentially improving both strength and corrosion resistance. However, the biocompatibility of alloying elements must be carefully considered, as some elements (eg, aluminum, certain rare earth metals) may raise toxicity concerns with prolonged exposure.^43^ High-purity Mg offers the advantage of avoiding such potential toxicities, but at the cost of reduced mechanical strength. Future iterations may benefit from carefully selected, biocompatible alloying elements combined with optimized surface treatments to achieve the balance of strength, degradation control, and safety required for maxillofacial applications.

Radiographically, HP-Mg implants exhibited lower radiodensity than titanium, as expected from their elemental composition.^44^ In our study, Mg plates were detectable on CBCT, albeit with reduced intensity compared to cortical bone, which may necessitate the development of specific CBCT protocols for effective follow-up. However, while the Mg hardware remained visible on CBCT, it was not detectable on plain radiographs. This contrasts with previous clinical reports in orthopaedics, where HP-Mg implants are readily traced on standard X-rays to monitor degradation postoperatively.^20^ This discrepancy may be attributed to the more delicate and slender geometry adopted in our design, which is much slimmer than the hardware typically used in orthopaedic procedures. Consequently, it was difficult, if not impossible, to accurately monitor the degradation of the Mg hardware in our study. Importantly, no obvious beam-hardening artifacts were observed from the Mg plates or screws, highlighting a significant advantage of using Mg for internal fixation.

Beyond biomechanical considerations, the requirements in oral and maxillofacial surgery are uniquely challenging compared to orthopaedic settings. The surgical field is continuously exposed to bacteria from the oral or nasal cavity and lacks the deep soft tissue envelope present in long bones.^31^^,^^32^ If early wound dehiscence or fistula occurs and is not promptly resolved, saliva and food particles may enter the wound and come into direct contact with the Mg hardware.^32^ Even in small quantities, this exposure can accelerate Mg degradation and lead to fixation failure. Such scenarios can provoke foreign body reactions and persistent discharge until the hardware is spontaneously expelled and the wound resolves. When bone union is hindered by wound exposure, inflammation, or inadequate mechanical stability, maxillary nonunion occurs, necessitating reoperation under general anaesthesia to remove granulation tissue and recreate a fresh bone contact surface for osteosynthesis.^33^ In summary, while HP-Mg shows promise, direct extrapolation from orthopaedic evidence may be misleading. The first application of HP-Mg in maxillofacial surgery underscores the innovation of this study and raises important questions for future research.

This pilot clinical observational study is limited by its small sample size, which restricts statistical analysis and precludes formal subgroup comparisons. As this was a first-in-field pilot feasibility study with no prior clinical data on HP-Mg fixation in maxillofacial surgery, a formal sample size calculation was not followed. The small sample size was based on practical considerations and precedent for early-stage device evaluation, aiming to provide initial safety and feasibility data to inform future larger-scale studies. Additionally, the HP-Mg plates and screws used were not specifically optimized for maxillofacial biomechanics, which may have contributed to the high complication rates. Further studies with optimized geometries and dimensions are necessary. Finally, surface modification strategies to enhance corrosion resistance were not evaluated; assessing such modifications in future studies may improve clinical outcomes.

In conclusion, HP-Mg plates and screws represent a promising and innovative biodegradable alternative for internal fixation in orthognathic surgery, demonstrating systemic safety, radiographic compatibility, and effective biodegradability. While some mechanical challenges and clinical variability were observed, these findings highlight valuable opportunities for further enhancement—such as the development and use of biocompatible Mg alloys, advanced surface modifications, and biomechanical designs tailored to maxillofacial conditions. With continued research and technological advancements, Mg has the potential to become a viable option for internal fixation in maxillofacial surgery.

## Data statement

Data will be available on request.

## Author contributions

All authors have participated in the research and article preparation. Wei-fa Yang has contributed to study design, implementation, and manuscript writing. Pak Chuen Patrick Tai has contributed to data collection and analysis. Ling Qin has contributed to study design and material preparation. Yiu Yan Leung has supervised the whole study. All authors commented on the manuscript and figures/table. All authors have approved the final manuscript.

## Conflict of interests

None disclosed.
